# The histone deacetylase inhibitor Romidepsin induces as a cascade of differential gene expression and altered histone H3K9 marks in myeloid leukaemia cells

**DOI:** 10.18632/oncotarget.26877

**Published:** 2019-05-28

**Authors:** Kathryn Clarke, Christine Young, Fabio Liberante, Mary-Frances McMullin, Alexander Thompson, Ken Mills

**Affiliations:** ^1^ Blood Cancer Research Group, Centre for Cancer Research and Cell Biology (CCRCB), Queen’s University Belfast, Belfast, United Kingdom; ^2^ Current address: Department of Haematology, Addenbrooke’s Hospital, Cambridge, United Kingdom; ^3^ Current address: MRC Human Genetics Unit, Institute of Genetics and Molecular Medicine, University of Edinburgh, Western General Hospital, Edinburgh, United Kingdom; ^4^ Current address: Ludwig Boltzmann Institute for Cancer Research, Wien, Austria; ^5^ Centre for Medical Education, School of Medicine, Dentistry and Biomedical Science, Queen’s University Belfast, Belfast, United Kingdom; ^6^ Current address: Division of Cancer and Stem Cells, Centre for Biomolecular Sciences, University of Nottingham, Nottingham, United Kingdom

**Keywords:** HDAC inhibitor, epigenetic, transcriptional regulation, myelodysplastic syndrome (MDS)

## Abstract

Myelodysplastic syndromes (MDS) are a heterogeneous, clonal haematopoietic disorder, with ~1/3 of patients progressing to acute myeloid leukaemia (AML). Many elderly MDS patients do not tolerate intensive therapeutic regimens, and therefore have an unmet need for better tolerated therapies.

Epigenetics is important in the pathogenesis of MDS/AML with DNA methylation, and histone acetylation the most widely studied modifications. Epigenetic therapeutic agents have targeted the reversible nature of these modifications with some clinical success. The aim of this study was to characterise the molecular consequences of treatment of MDS and AML cells with the histone deacetylase inhibitor (HDACi) Romidepsin.

Romidepsin as a single agent induced cell death with an increasing dose and time profile associated with increased acetylation of histone H3 lysine 9 (H3K9) and decreased HDAC activity. Gene expression profiling, qPCR, network and pathway analysis recognised that oxidation-reduction was involved in response to Romidepsin. ROS was implicated as being involved post-treatment with the involvement of TSPO and MPO.

Genomic analysis uncoupled the differences in protein-DNA interactions and gene regulation. The spatial and temporal transcriptional differences associated with acetylated, mono- and tri-methylated H3K9, representative of two activation and a repression mark respectively, were identified. Bioinformatic analysis uncovered positional enrichment and transcriptional differences between these marks; a degree of overlap with increased/decreased gene expression that correlates to increased/decreased histone modification. Overall, this study has unveiled a number of underlying mechanisms of the HDACi Romidepsin that could identify potential drug combinations for use in the clinic.

## INTRODUCTION

Myelodysplastic syndromes (MDS) are a heterogeneous group of clonal haematopoietic disorders characterised by bone marrow failure and disease-associated anaemia, thrombocytopenia and neutropenia. Concomitant leukemic transformation for approximately one third of MDS patients, which is more difficult to treat, will occur resulting in truncated survival [1, 2].

The majority of patients are elderly, >60 years, with reduced performance status, other co-morbidities and ineligible for transplantation. High-risk patients, who are ineligible for transplantation, can be treated with intensive chemotherapy (IC) or treatment with low-dose cytarabine (LDAC) [3]. However, neither has the ability to consistently prolong survival in high-risk MDS with a similar outcome to treatment with best supportive care [4–6]. This highlights the need for better tolerated and more molecularly targeted therapies for these patients. Epigenetic therapies such as DNA methyl transferase inhibitors (DNMTi) and histone deacetylase inhibitors (HDACi) have been used in clinical trials as single agents or in combination with other epigenetic drugs, chemotherapy or other novel molecular therapies [7–9].

HDACs regulate cell cycle progression, survival and angiogenesis and therefore are a potential therapeutic target in a variety of malignancies [10]. HDACi are used to shift the equilibrium of histone and non-histone protein acetylation resulting in a modification of the epigenetic landscape and changes in the expression, functionality and localisation of non-histone proteins [11, 12] including the tumour suppressor p53 [13]. Six distinct groups of HDACi have been developed each with different structural integrities and selectivity profiles as pan inhibitors (e.g. Vorinostat) or with more specific inhibitory effects on selected HDACs (e.g. Entinostat). One such specific HDACi used for the inhibition of Class I HDACs is Romidepsin, a bicyclic tetra-peptide that has been FDA approved for the treatment of cutaneous T-cell lymphoma and undergoing clinical trials for the treatment of other malignancies [14–16]. Furthermore, Romidepsin has been identified as a potential therapeutic for MLL-rearranged infant acute lymphoblastic leukemia [17].

The mechanism of action of Romidepsin remains to be fully elucidated and this study has investigated the underlying mechanism of action of Romidepsin at the cellular and molecular level to identify potential therapeutic targets for further combination therapies.

## RESULTS

### Romidepsin results in a reduction in cell viability, altered protein acetylation and changes in cell cycle

**Figure 1:**
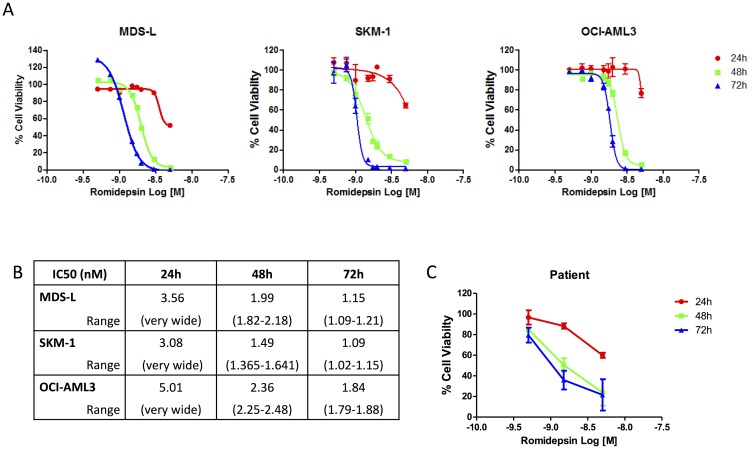
Dose dependent response of Romidepsin in cell lines and patient samples. (**A**) OCI-AML3, SKM-1 and MDS-L cell lines treated with a dose range of Romidepsin with cell viability being assessed using the Cell Titer Glo^®^ Assay at 24, 48 and 72 hours. IC50 values were determined based on cell line and time using GraphPad Prism software. (**B**) IC50 (nM) and range for Romidepsin for the three cell lines at each time point (24 hr, 48 hr and 72 hr). (**C**) Romidepsin treated patient samples over 24, 48 and 72 hours showing dose and time dependent reduction in cell viability following treatment as measured using Cell Titer Glo^®^.

A dose response was performed to identify the pattern of response on cell viability following treatment with Romidepsin. The IC50 (inhibiting 50% of proliferative capabilities) was deduced following analysis of an increasing range of doses from 0.1–5 nM Romidepsin (Figure 1A and 1B). The sensitivity to the drug was in the nM range for all three cell lines, with 72 hour IC50 values between 1–1.8 nM. Dose responses were conducted on two AML patient samples (LREC: 08/NIR1/9) which also showed a dose and time dependent decrease in viability of these cells post-Romidepsin treatment (Figure 1C).

**Figure 2:**
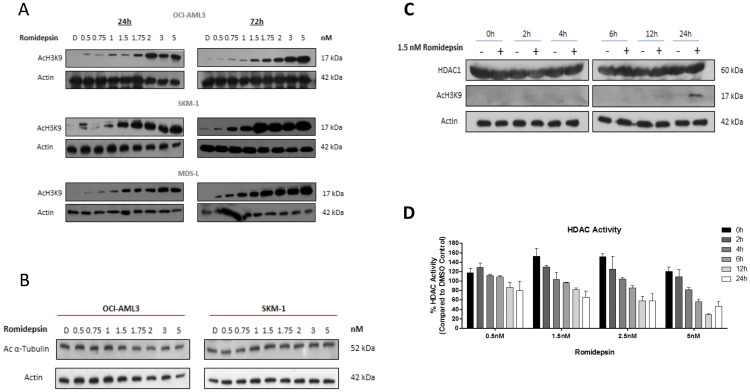
Romidepsin effects histone and protein acetylation. (**A**) Western blot analysis of OCI-AML3, SKM-1 and MDS-L cells for H3K9ac following increasing concentrations of Romidepsin at 24 and 72 hours showing that all the cell lines were susceptible to a dose dependent increase in levels in acetylation by 24 hours and with lower doses by 72 hour following treatment. (**B**) Protein expression analysis by western blot measuring levels of acetylated alpha tubulin in OCI-AML3 and SKM-1 at 24 hours. Romidepsin is a selective HDAC inhibitor for HDACs 1 and 2, and 4 and 6 to a lesser extent. Acetylated α-Tubulin is a substrate of HDAC 6. Treatment with Romidepsin had a limited effect on HDAC 6 does not result in an increase in acetylation of α-Tubulin. (**C**) Protein expression using western blot analysing levels of HDAC 1 and H3K9ac following treatment with 1.5 nM Romidepsin showing no change in HDAC1 protein expression after treatment over a 24 hour time period, but that by 24 hour there is an increase in acetylation of H3K9 with 1.5 nM Romidepsin. (**D**) OCI-AML3 cells were treated with varying doses of Romidepsin over a 24 hour time frame. Class I and II HDAC activity levels were measured using the HDAC-Glo™ I/II assay and demonstrated a reduction in HDAC activity commensurate with an increase in acetylation. Results are representative of a biological and technical triplicate and as a percentage of DMSO control.

As it has been documented that Romidepsin preferentially results in acetylation of lysine residues on histone 3 (H3) over that of histone 4 (H4) [18], the levels of acetylation on H3 lysine 9 (H3K9) were measured following treatment with increasing doses of Romidepsin. Figure 2A shows the increase in acetylation of H3K9 following treatment with 0.5 to 5 nM at 24 hours, with increased acetylation also observed with the lower doses by 72 hours. Romidepsin preferentially inhibits HDACs 1 and 2; if HDAC 6 was inhibited the levels of acetylation on non-histone proteins such as α-Tubulin should also increase. However, as shown in Figure 2B, no increase in acetylation of α-Tubulin was seen, therefore Romidepsin does not inhibit the activity of HDAC 6 in this instance.

Due to the increases in acetylation of histone proteins observed at 24 hrs (Figure 2A), a candidate dose of 1.5 nM was mainly used for subsequent studies. This dose resulted in an increase in acetylation but not in an overt amount of cell death that could be attributed to cytotoxicity. As demonstrated in Figure 2C, this dose had the potential to induce marked acetylation by 24 hours whilst sparing levels of HDAC 1 protein. However, this also implies that a reduction in levels of HDAC 1 is not necessary to result in increased acetylation but potentially blocks activity of that HDAC. To explore this further, the levels of HDACs specific to Class I and II in OCI-AML3 cells were investigated across a range of Romidepsin doses (Figure 2D). A gradual reduction in HDAC activity with 0.5 and 1.5 nM Romidepsin at time points up to 24 hrs was seen; whilst higher doses display a greater reduction in activity as early as 12 hrs. This correlated with the increased in acetylation of H3K9 in Figure 2C.

HDAC inhibitors have been linked with altering the cell cycle and whilst there was an increase in the Sub G0 population with an associated reduction in all other phases of the cell cycle with increasing doses, lower doses had little to no effect on cell cycle. No evidence of apoptotic behaviour was observed at 0.5 nM, with modest levels detected at 1.5 nM with cells entering early phase apoptosis by 48 hr and late phase by 72 hrs (Supplementary Figure 1). The higher dose of 5 nM results in detection of early phase apoptosis by 24 hr and 80% apoptosis by 72 hr.

### Gene expression profiling following Romidepsin treatment

**Figure 3:**
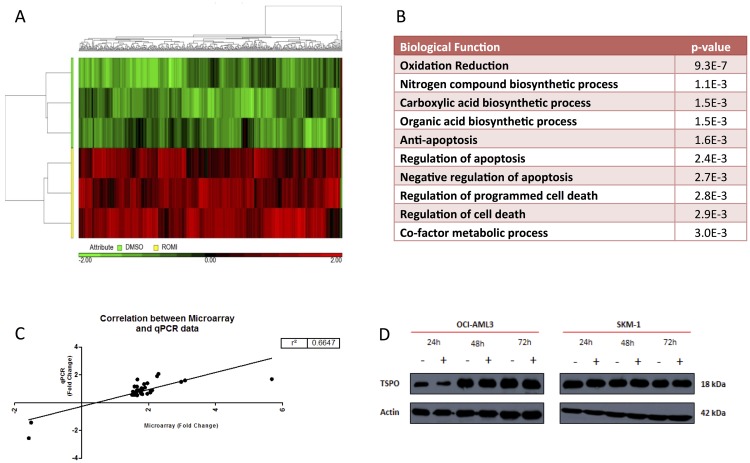
Unsupervised hierarchical clustering heat-map of 487 differentially expressed probe-sets between DMSO and Romidepsin treated samples in SKM-1 cells. (**A**) Heat-map displaying a visual representation of differentially expressed genes between control group in green, along Y axis, and Romidepsin treatment in yellow on Y axis. Unsupervised hierarchical clustering was performed which generated these clusters based on treatment groups and on genes which are up and down-regulated. A clear separation between control and treatment group is visible with the vast majority (99%) of genes being up-regulated (red) following Romidepsin treatment. (**B**) Most significantly up-regulated processes as identified by DAVID following Romidepsin treatment on SKM-1. (**C**) Correlation of microarray log(2) intensity values and Ct values from real-time PCR of the 39 candidate genes. (**D**) Increased protein expression of TSPO following treatment with 1.5 nM Romidepsin over 72 hours in OCI-AML3 and SKM1 cells.

Affymetrix HG U133 plus 2.0 arrays were used to perform gene expression profiling on SKM1 cells treated with DMSO or 1.5 nM Romidepsin. The candidate dose of 1.5 nM Romidepsin was selected based on the data collected in the previous sections, including a minimal effect on cell viability but with the ability to induce molecular effects such as an increase in acetylation concomitant with a decrease in HDAC activity. Stringent criteria were applied following an ANOVA of all genes which included the application of an unadjusted *p*-value = <0.05 and a fold change of ≤ and ≥ 1.5. These criteria were applied to identify significantly differentially expressed genes and those which are deemed to have changed to a biologically relevant degree. This generated a gene list consisting of 487 probe-sets that were significantly differentially expressed between control and treated sets; the 487 probe-sets represented 442 genes. Of these 487 differentially expressed probe-sets, 484 (99%) were significantly up-regulated versus only 3 (1%) down-regulated (Figure 3A). Pathway analysis (Figure 3B) using the online tool DAVID identified oxidation reduction as the most significantly enriched pathway, followed by bio-synthetic processing and regulation of apoptosis. A total of 38 (7.8%) genes from the gene list were validated by RQ-PCR (Figure 3C) and these were associated with oxidation and reduction including *MARC1* and *2*, *WWOX* and *ADI1* with a large number of cytochrome family members also included. Genes involved in nitrogen and carboxylic acid biosynthetic processing included *PAPSS1, ATP6V0E2, AGPAT1* and *CD74*, whilst those involved in regulation of apoptosis consisted of *ADAMTSL4, ATG5 and TRAF5*. Gene ontology (GO) enrichment scores for biological processes and molecular functions are shown in Supplementary Figure 2. Further network connectivity was performed using the online database STRING to identify known and predicted protein-protein interactions. Supplementary Figure 3 shows networks identified in STRING which were then imported into Gephi for visualisation. TSPO was at the centre of a highly connected node with a high degree of betweenness centrality, implying this protein may be essential for the response to Romidepsin (Figure 3D). The activation of TSPO either by ligand or cholesterol can result in the release of ROS from the outer mitochondrial membrane with, as yet, unknown consequences on downstream effectors [19]. Interestingly, other nodes were centred on an increased expression of *FYN and CBL*, both of which are associated with ROS activation.

### Histone H3 profiling using ChIP-Seq following treatment with Romidepsin

**Figure 4:**
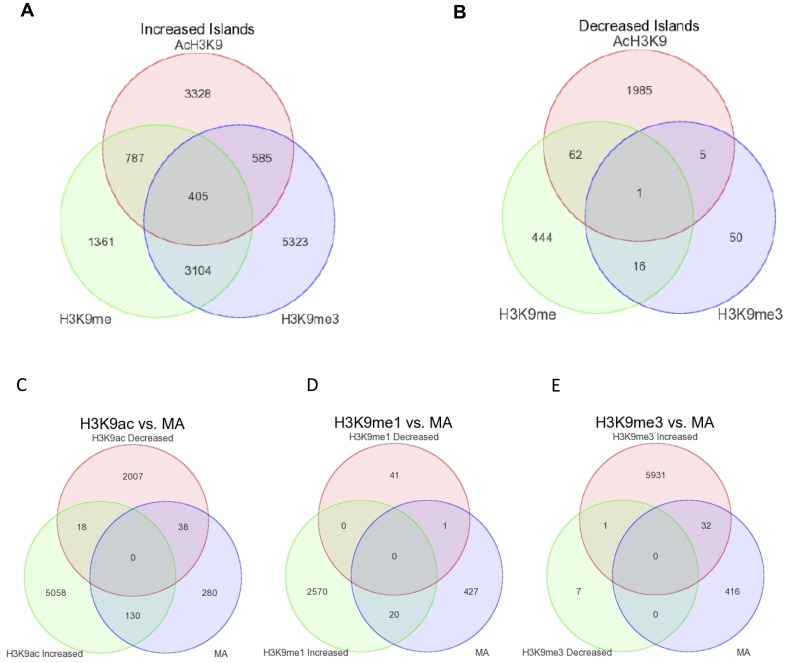
Overlap in genes regions associated with increased and decreased islands across all three histone marks and differentially expressed genes. Gene regions associated with increased and decreased islands generated in SICER were imported to Partek for overlap analysis. Overlap of positional enriched regions showing increased (**A**) or decreased (**B**) acetylation/methylation for the three marks. Overlap of differential histone marks and differentially expression genes by microarray for H3K9ac (**C**), H3K9me (**D**) and H3K9me3 (**E**).

The known Romidepsin target Histone H3 was selected analysis by chromatin immunoprecipitation sequencing (ChIP-seq) profiling focusing on lysine 9 modifications; H3K9ac, H3K9me and H3K9me3. This allowed for the analysis of two activation marks (H3K9ac and H3K9me) alongside one repression mark (H3K9me3) following 24 hour treatment with 1.5 nM Romidepsin on SKM-1 cells. The acetylation of TSPO, which we had shown to be differentially expressed, was used as a marker of successful enrichment of H3K9ac (Supplementary Figure 4). The software SICER 1.1 was used to generate a list of genomic co-ordinates that showed increased and decreased enrichment for each mark following Romidepsin treatment: 21,940 peaks showed increased H3K9ac with only 2,453 decreased; H3K9me was increased at 16,734 peaks and decreased at 1,094; and increased H3K9me3 was seen at 30,024 peaks with only 352 peaks showing a decrease. Further positional enrichment was done using the criteria for: H3K9ac of ± 1000 bp from the transcriptional start site and not in the gene body; H3K9me within the gene body; and H3K9me3 of within –2000 to +200 bp of the TSS and within the gene body (Supplementary Figure 5). The overlap of gene regions using the positional enrichment criteria for three marks reduced the number of relevant increased and decreased gene regions across all three histone marks and suggested that there was some degree of overlap within the increased islands cohort (Figure 4A), but very little overlap with the decreased islands (Figure 4B).

Further, we overlapped the increased and decreased islands for each mark, using the positional enrichment criteria with the 4,488 differentially expressed genes identified from the microarray analysis. Reassuringly, there were no genes that were common to both increased and decreased islands for any of the mark that overlapped with microarray data. Interestingly, the group with the largest proportion of overlap with the microarray data (Figure 4C–4E) is with that of increased acetylation whereby 130 genes overlap (Supplementary Table 1; Supplementary Figure 6).

## DISCUSSION

A plethora of epigenetic modifying drugs are currently being examined both in the pre-clinical and clinical setting. DNA demethylating drugs, such as Azacitidine, have proven successful in the treatment of high-risk MDS and AML after receiving FDA approval in 2004. Although harbouring the potential to extend survival by 9.5 months, little is known about the precise mechanism of action that is involved eliciting response following treatment. Another of the new generation epigenetic modifying drugs which has also been used in a clinical setting is the histone deacetylase inhibitor (HDACi) Romidepsin. It received FDA approval in 2009 for the treatment of cutaneous and peripheral T cell lymphoma [15], however little is known about its underlying mechanism of action. As single agents, HDACi do not produce the expected survival responses in patients and so with that, it is hypothesised that combinations of these epigenetic drugs may induce synergistic effects [11, 12] and increase survival probability in patients.

This study aimed to extrapolate the underlying mechanism of action of the HDAC inhibitor Romidepsin with the potential of identifying differentially expressed genes, novel pathways and downstream effects that could be explored as targets for rational combinations for potential use in the clinic.

Romidepsin has been used in patients with cutaneous T-cell lymphoma (CTCL) and peripheral T-cell lymphoma (PTCL), however, very little has been explored in the context of MDS/AML [20] and response to the drug has been termed sub-optimal for use as a single agent [21]. Furthermore, a high-throughput screening revealed Romidepsin enhanced the activity of a key component of infant ALL therapy, cytarabine (ARAC) *in vitro* and *in vivo* [17]. It has been used in the treatment of MDS/AML as a Phase I clinical trial (ROMAZA, UKCRN Study ID: 15082) in combination with Azacitidine. Therefore, as limited pre-clinical data was available using Romidepsin in this setting, we have assessed the cellular and molecular effect in MDS/AML cell line models. A dose and time-dependent decrease in cell viability was observed with a subsequent increase in the proportion of apoptotic cells with a related increase in the proportion of cells in sub G0. There was a correlation with an increase in protein expression of acetylated histone H3K9 with increasing concentrations of Romidepsin and a preceding decrease in HDAC activity at earlier time-points. It has been previously been recognized that HDACIs induce acetylation of histone H3 at lower concentrations lower than those that induce cell death [18]. The increase in acetylation was independent of any observable differences in HDAC1 protein or gene expression. Acetylation of the cytoplasmic protein α-Tubulin remained unaffected following treatment; however this was an expected observation as Romidepsin is a selective HDAC inhibitor that does not target HDAC6, the binding partner of α-Tubulin. Romidepsin treatment contributes to these associated changes in cell cycle and has the potential to alter the expression of p21 [22] and the cell surface marker CD11b on OCI-AML3 and SKM-1 cells (data not shown).

Transcriptional analysis of 1.5 nM Romidepsin after 24 hrs identified 487 differentially expressed probe sets of which 484 were up-regulated compared to only 3 down-regulated. These 487 probe-sets represent 442 genes. Pathway and network analysis identified oxido-reductase activity as the most significantly enriched pathway with hubs forming around genes associated with this pathway. The induction of oxidative injury has been seen with other HDACis [23]. One such gene in our pathway that was strikingly poignant was TSPO [24]. This was biologically significantly up-regulated following treatment with Romidepsin and also appeared to be central in the response to treatment. Network analysis also highlighted it as having a high degree of connection as well as forming a bottleneck–often deemed more biologically relevant than massive up-regulation of a single gene. TSPO is located in multiple sites, including haematopoietic and lymphatic cells and has multiple functions [24]. It has since been shown to be a cholesterol-binding protein with the ability to transport cholesterol from intracellular stores to the mitochondria. It has also been linked with ROS production and one theory is that external stimulus will alter TSPO activity and ultimately result in the opening of mitochondrial membrane pores [25]. This may lead to the production of ROS which can impact on several pathways downstream, but that an immediate release of cytochrome C through membrane pores such as BAX will initiate mitochondria-mediated apoptosis. Although further investigation will be required, ROS was implicated in other ways in this study and in the literature as being associated with HDACi treatment [26, 27].

Our next aim was to explore the effect that Romidepsin had on histone H3 activation/repression status whilst integrating this with the differentially expressed genes. Three histone marks, acetylated, monomethylated and trimethylated H3K9, were chosen as representative of two activation marks and a repressive mark respectively. The integration of the transcriptional program for this setting provided a more comprehensive view of what is being differentially regulated on H3K9 [28]. Uniquely enriched peaks were identified in the normalised Romidepsin samples using SICER and these peaks were then analysed to ascertain their positional enrichment prior to peak annotation. From here, gene lists were produced that were specific to each individual mark based on their positional enrichment. Overlap was identified between all three marks in both increased and decreased islands, signifying that there is the potential for genes to harbour enrichment of all three marks which could be as a result of cells not all being synchronised to the same phase of the cell cycle. This could also represent a somewhat dominant effect of certain marks on their ability to exert an active or repressive effect, but that will require further investigation to fully elucidate this potential. There was also a degree of overlap observed with increased and decreased enrichment on individual marks, again something that could be as a result of cells not being synchronised. Integration with microarray data suggested that there were 130 genes associated with increased gene expression and enrichment of H3K9 acetylation, implying activation of these genes. Only 32 genes were associated with increased trimethylation on H3K9 and gene expression, indicating a role in repression of these genes following treatment with Romidepsin. Whilst it is recognised that further analysis and investigation of the genes and networks is required, this study has suggested that there are potential Romidepsin induced targets, such as ROS, for rationale combination therapies for AML/MDS patients.

## MATERIALS AND METHODS

### Materials

Romidepsin (Celgene Inc., USA) was dissolved in DMSO at a stock concentration of 100 mM; however the final DMSO concentration for *in vitro* assays was always at 0.1%.

### Cell lines

OCI-AML3, MDS-L and SKM-1 cells (Deutsche Sammlung von Mikroorganismen und Zellkulturen GmbH, Braunschweig, Germany), were maintained in RPMI-1640 media supplemented with 10% fetal calf serum, 1% Pen/Strep and incubated in 5% CO_2_ at 37° C.

### Viability, caspase activity assays

Cell viability and caspase activity was determined using the CellTitre-Glo luminescent viability kit and caspase Glo assays (Promega, Madison, USA) as described in the manufacturer’s instructions.

### FACS analysis

To assess cell cycle changes, cells were harvested and re-suspended in 70% ice-cold ethanol overnight followed by staining with propidium iodide mix (40 μg/mL PI, 2 ng/ml RNase A). BD FITC Annexin V apoptosis detection kit (BD Biosciences, California, USA) was used according to the manufacturer’s instructions to assess apoptosis. Samples were analysed using LSRII and BD FACSDiva software.

### Western blot analysis

Cells were washed and lysed using RIPA buffer (50 mM Tris (pH 7.4), 150 mM NaCl, 5 mM EDTA, 1% triton X (x100) and 0.1% SDS containing protease inhibitor cocktail tablets (Roche Products Ltd, Welwyn Garden City, UK) and phosphatase inhibitors (sodium fluoride and sodium orthovanadate). Protein concentration was quantified (Pierce BCA Protein Assay kit, Thermo Fisher Scientific, USA). Total lysates were prepped by adding 10X loading buffer to 30 μg of total lysate, denatured for 5 minutes at 95° C. Lysates were then electrophoresed on various percentage Tris Glycine gels and immunoblotted using anti-actin (Sigma, UK), anti-acetylated tubulin (Cell signalling, Massachusetts, USA), anti-hyperacetylated histone H3 (Merck Millipore, UK), anti-HDAC1 and anti-TSPO (Abcam, UK) antibodies.

### Quantitative real-time PCR

RNA was extracted using the RNeasy^®^ kit (Qiagen, Crawley, U.K.) and was eluted using RNase-free water and experiment was performed in accordance with manufacture’s protocol. The High Capacity Reverse Transcription Kit (Applied Biosystems, Life Technologies, UK) was used for the conversion of RNA to cDNA in accordance with manufacturer’s instructions.

Sybr-Green real time quantitative PCR was performed using the 7900HT Real-time PCR system under optimal cycling condition. Primers used for mRNA quantification were designed in-house using Primer-Blast and purchased from Eurofins Genomic (Eurofins Genomic, Ebersberg, Germany). Primer sequences used can be found in Supplementary Table 2. Validation of microarray studies was carried out using RNA matched samples to those used for hybridisation to the Affymetrix arrays.

Gene expression is shown as fold change of the control treatment and normalised to the endogenous controls. The Ct value for each gene was normalised to β-Actin and 18S endogenous controls (ΔCt), ΔCt was then normalised to the treatment control (ΔΔCt), and the ΔΔCt was used to calculate gene expression changes as a fold change over control.

### Analysis of gene expression by microarray

RNA was extracted from biological triplicate samples using the RNeasy kit. A maximum of 8 μg of total RNA was processed to cRNA and hybridised to Affymetrix Human Genome U133 Plus 2.0 Arrays using an Affymetrix GeneChip Hybridisation Oven and following the protocol outlined by Roche Diagnostics (Burgess Hill, UK). Arrays were then stained using an Affymetrix GeneChip Fluidics Station 450 and scanned using the Affymetrix GeneChip Scanner 3000 5G. Analysis of data was performed using Partek Genomic Suite Software (Partek Incorporated, Missouri, USA). Following CEL file importation to Partek Genomic Suite, data were analysed using a Robust Multi-array (RMA) analysis to correct for background, quantile normalise and summarise the probe set intensity into expression measurements.

### ChIP-qPCR

A total of 1 × 10^6^ cells per IP were harvested from drug treated cells and fixed in 1% formaldehyde. Cells were washed and chromatin was isolated; fixed samples were suspended in LB1 buffer (50 mM Hepes-KOH, pH 7.5, 140 mM NaCl, 1 mM EDTA, 10% glycerol, 0.5% IGEPAL, 0.25% Triton X-100) for 10 minutes, pelleted and suspended in LB2 buffer (10 mM Tris-HCl, pH8, 200 mM NaCl, 1 mM EDTA, 0.5 mM EGTA) for 5 minutes, pelleted and suspended in LB3 buffer (10 mM Tris-HCl, pH8, 100 mM NaCl, 1 mM EDTA, 0.5 mM EGTA, 0.1% NA-deoxycholate, 0.5% N-laurylsarcosine). Cells were sonicated for 9 minutes to obtain fragments of between 200–500 bp. Following sonication, 10% triton X-100 was added and chromatin was snap frozen for chromatin immunoprecipitation studies. To pre-block beads and conjugate to antibody; 30 μL beads per IP were washed and blocked overnight at 4° C in sterile 0.5% BSA:PBS. Chromatin was immunoprecipitated with chosen antibodies (anti-histone H3 (acetyl K9), Anti-histone H3 (mono methyl K9) and Anti-histone H3 (tri methyl K9) (Abcam, UK) and eluted using 20 μL of elution buffer (50 mM Tris pH8, 10 mM EDTA, 1% SDS). Chromatin was purified using the QIAquick PCR purification kit (Qiagen) as described in the manufacturer’s instructions.

### ChIP-Seq

Chromatin Immunoprecipitation followed by DNA sequencing (ChIP-Seq) was performed. The process is described by Schmidt *et al*. in 2009 [29] and in brief involves cross-linking proteins to DNA followed by fragmentation of these complexes to 200–500 bp. Upon confirmation of appropriately sized fragments, sheared chromatin is then bound to an antibody of choice to be immuno-precipitated before cross-links are reversed with proteinase K and DNA is purified. The eluted DNA then underwent adapter ligation and library preparation with a size selection stage prior to being sequenced using an Illumina^®^ Next-Seq 500.

### Statistical analysis

All statistical analysis was performed using Graphpad Prism version 5 for Windows (California, USA). All statistical tests were performed on at least a biological replicate of 3.

### Analysis software and packages

Partek^®^ Genomic Suite™ Software was used for analysis of all microarray data for visualisation and exploratory analysis. STRING v 10 - Search Tool for the Retrieval of Interacting Genes was used for network analysis of gene lists produced (127) and Gephi v 0.8.2-beta used for network visualisation and manipulation. DAVID v 6.7 - Database for Annotation, Visualization and Integrated Discovery was the web-accessible program used for all pathway analysis (128,129). R version 3.2.0 was used (https://www.r-project.org) in the background of several of the packages used in ChIP-Seq analysis. ChIP-Seq packages included; Trim Galore! v 0.4.0, FASTQC v 0.11.3, Bowtie2 v 2.2.5, SAMTools v 1.2, BEDTools v 2.17.0, SICER v 1.1, PASPT v 1.0, CEAS v 0.9.9.7 and IGV v 2.2.57. All ChIP-Seq packages were run on Linux. 82.

## SUPPLEMENTARY MATERIALS




