# Supplementary material for: The histone deacetylase inhibitor Romidepsin induces as a cascade of differential gene expression and altered histone H3K9 marks in myeloid leukaemia cells
Source: Oncotarget. 2019 May 28;10(37):3462–71. doi: 10.18632/oncotarget.26877 (PMC6544403; doi:10.18632/oncotarget.26877)
Supplement: Supplementary file 1 [file oncotarget-10-3462-s001.pdf]

The histone deacetylase inhibitor Romidepsin induces as a cascade of differential gene expression and altered histone H3K9 marks in myeloid leukaemia cells

SUPPLEMENTARY MATERIALS

REFERENCES

1. Yu H, Kim PM, Sprecher E, Trifonov V, Gerstein M. The importance of bottlenecks in protein networks: correlation with gene essentiality and expression dynamics. PLoS Comput Biol. 2007; 3:e59. https://doi.org/10.1371/journal.pcbi.0030059. [PubMed]

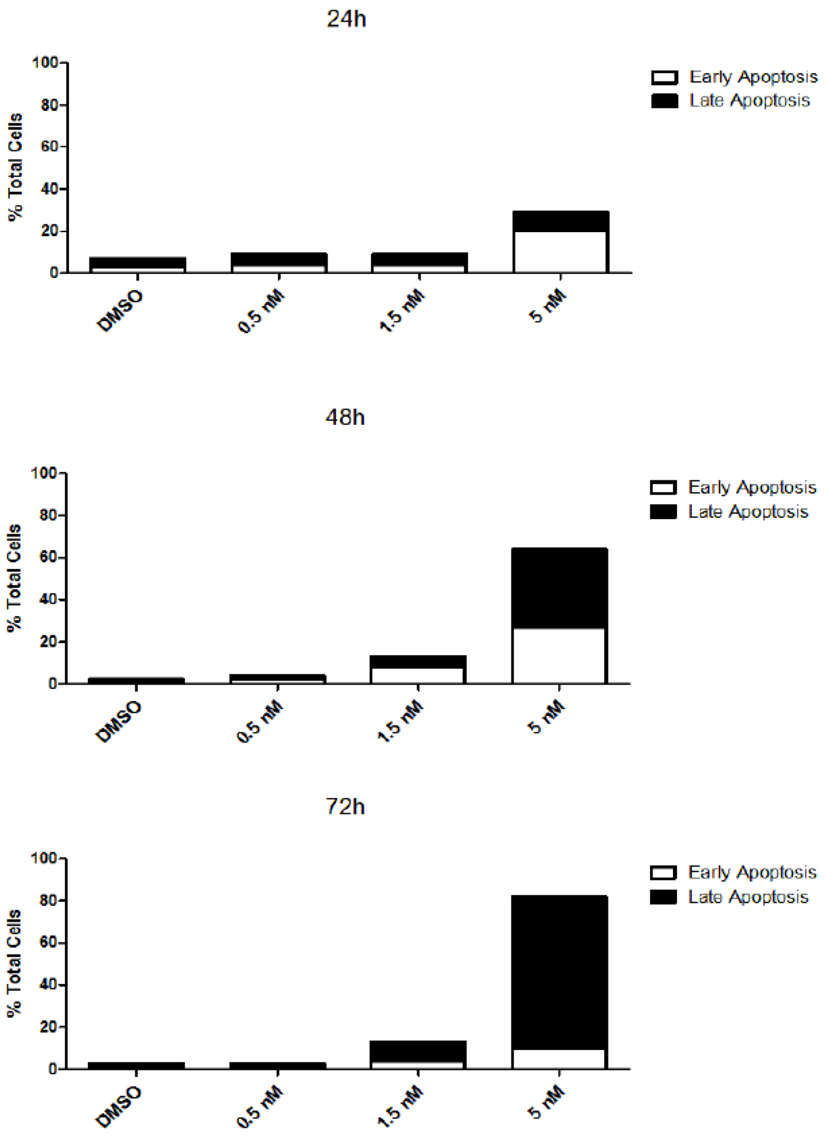

Supplementary Figure 1: Tracking of early and late apoptosis in SKM-1 cells following treatment with Romidepsin. Annexin V and PI staining used to determine stages of apoptosis following treatment with DMSO, 0.5 nM, 1.5 nM and 5 nM Romidepsin. Observed is an increase in the percentage of cells entering into late apoptosis as time and dose increase.

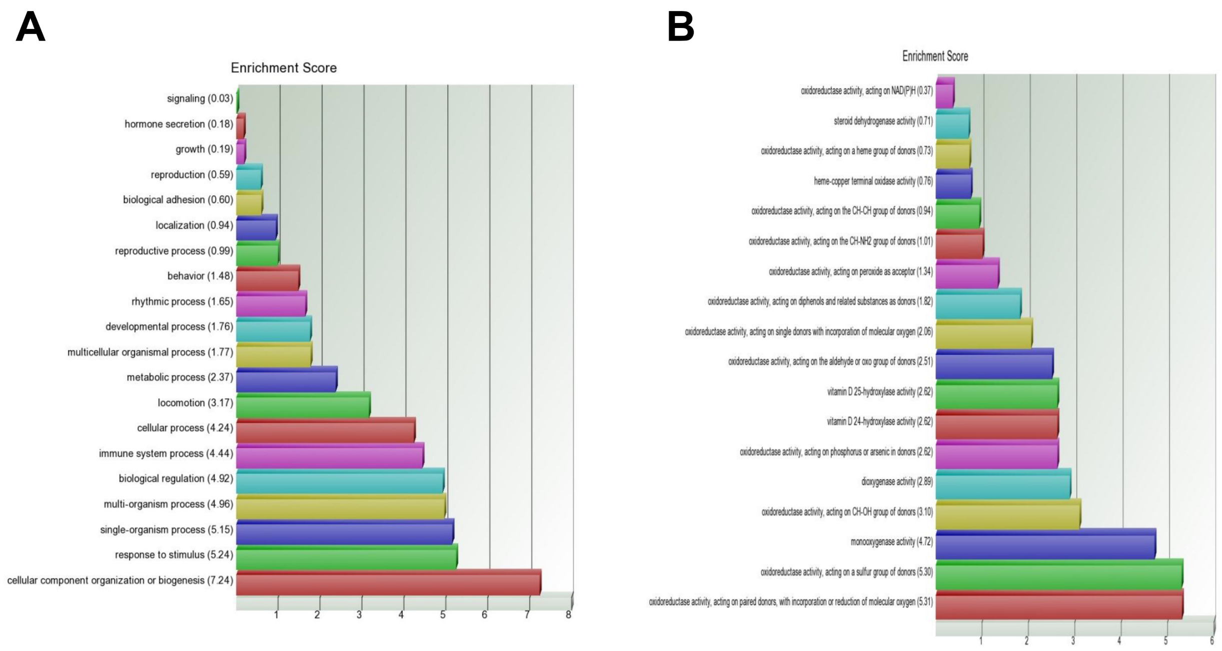

Supplementary Figure 2: Gene Ontology (GO) processes analysis. (**A**) GO biological processes analysis identifying that cellular organisation and biogenesis was the top most enriched process followed by response to stimulus with immune system process also affected. (**B**) GO molecular functions identified oxidoreductase activity as the most enriched pathway following treatment with an enrichment score of 5.31 with other related oxidoreductase activities being affected.

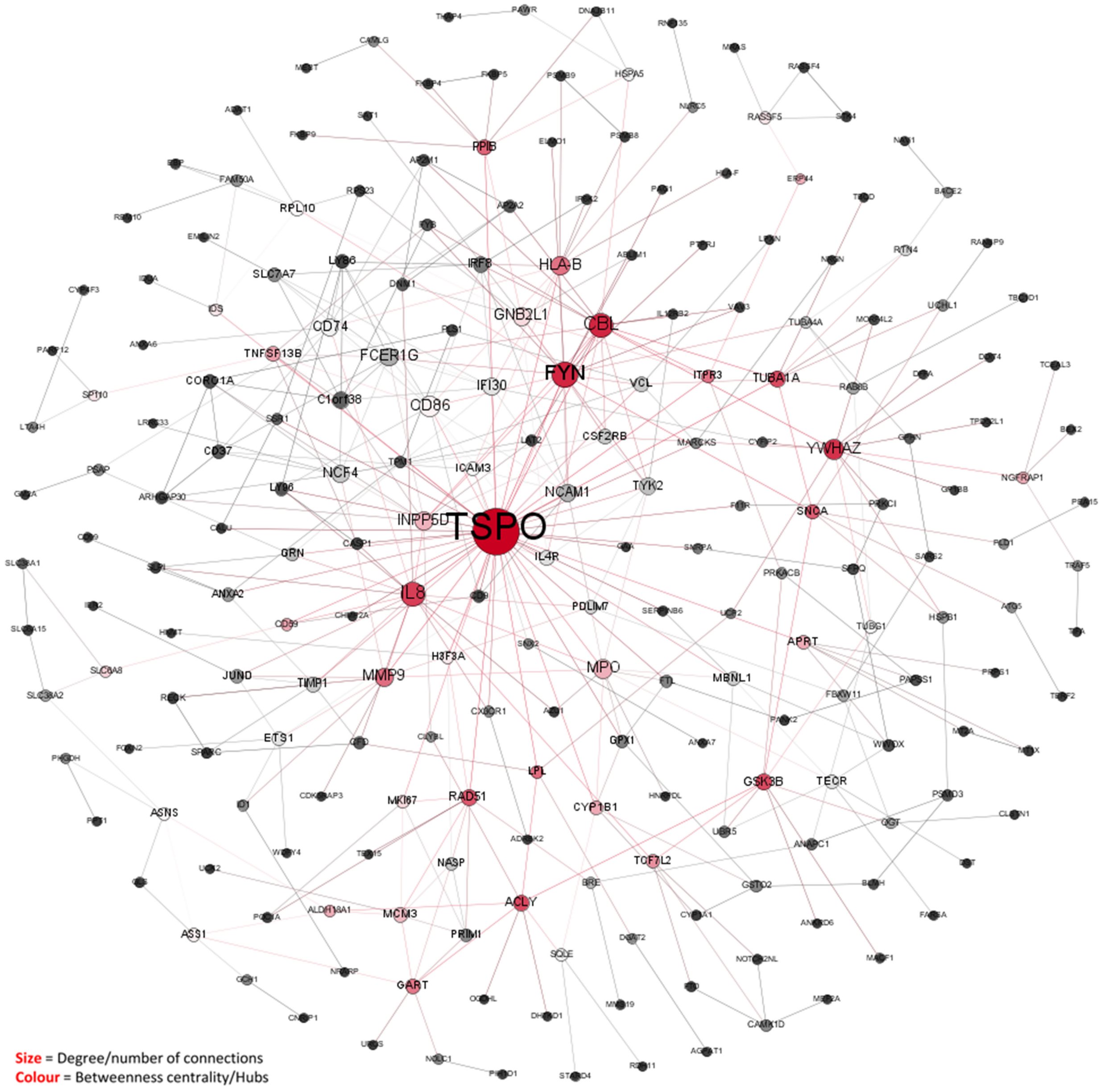

Supplementary Figure 3: STRING Known and predicted protein–protein interactions, followed by GEPHI configuration. Network analysis of 487 genes associated with differential gene expression following Romidepsin treatment. The 487 gene list was imported to STRING which enabled the formation of network matrices. These matrices could then be uploaded to a visualisation software package, Gephi 0.8.2-beta (http://gephi.org/), which enabled for the manipulation of shapes and colours to reveal and emphasise the importance of particular genes associated with hubs and bottlenecks based on their betweenness centrality (indication of a nodes centrality to a hub) and degree on connections. Bottlenecks are described as being proteins with a high degree of betweenness centrality and are believed to be "key connector proteins with surprising functional and dynamic properties" and are said to be more likely essential proteins within the network [1]. Criteria applied here included size of the node to depict degree/number of connections, whilst colour denotes betweenness centrality i.e. the larger the node, the more connected the protein is, and the more red the node, the higher the betweenness centrality.

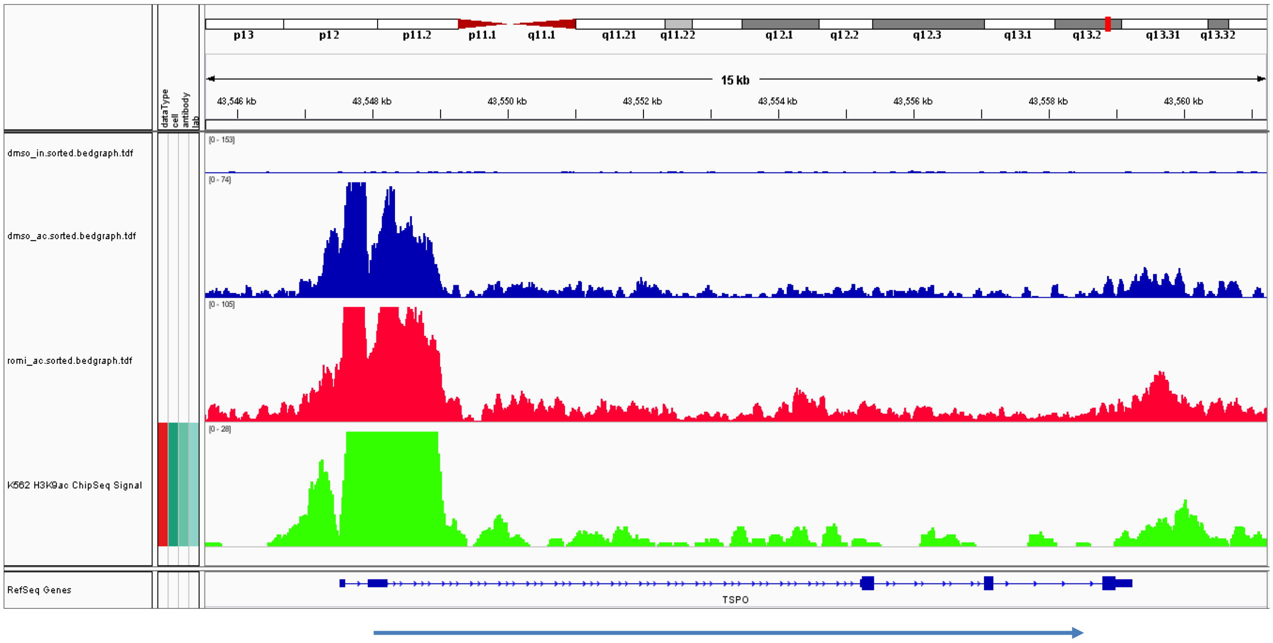

Supplementary Figure 4: IGV tracks showing acetylation levels at the promotor of TSPO. TSPO was used as a positive control as it had been shown to be acetylated at the promoter in the closely related haematopoietic cell line K562 prior to sequencing. As can be seen in the tracks above, this was also the case in our samples (blue = SKM-1 cells treated with DMSO for H3K9ac, red = SKM-1 cells treated with Romidepsin for H3K9ac, green = control K562 H3K9 acetylation track from IGV) for validating TSPO as a positive control. Input sample shows little to no signal.

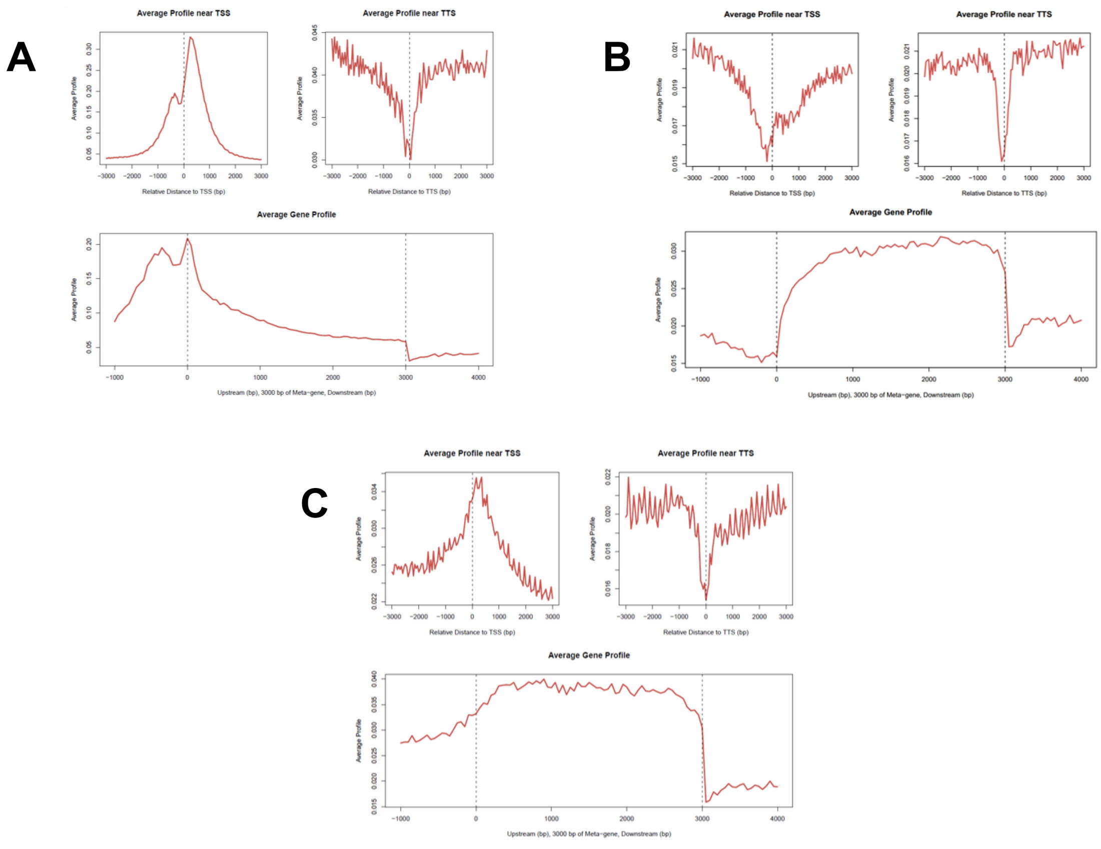

Supplementary Figure 5: Cis-regulatory Element Annotation System (CEAS) profiles of TSS, TTS and average gene profile of AcH3K9, H3K9me and H3K9me3 in Romidepsin treated samples. CEAS profiles showing (**A**) AcH3K9, (**B**) H3K9me and (**C**) H3K9me3. Both (**A**) and (**C**) display peaks around the transcriptional start site (TSS) and dips at the transcriptional termination site (TTS), whilst (**B**) portrays a dip at the TSS. Acetylation appears to taper off across the gene body, whilst methylation in both mono and tri form maintain high levels of enrichment throughout gene body.

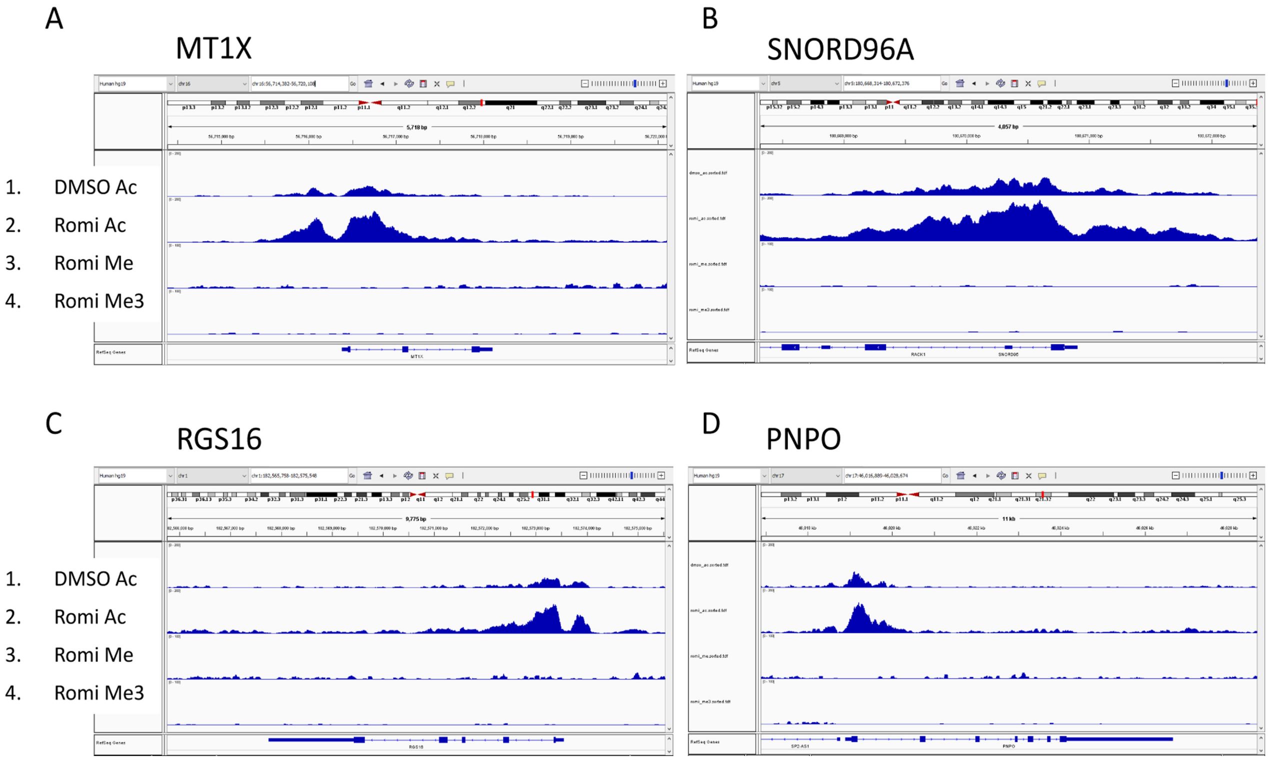

Supplementary Figure 6: Examples of IGV tracks showing increased acetylation levels of genes with increased differential expression. Increased Acetylation marks in (**A**–**D**). Row 1 (top) is DMSO treated cells for Acetylation marks, row 2 is Romidepsin treated cells for acetylation marks; row 3 is Romidepsin treatment for mono-methylation and row 4 (bottom) is tri-methylation following Romidepsin treatment.

Supplementary Table 1: 130 genes showed an overlap between microarray data and increased acetylation. See Supplementary_Table_1

Supplementary Table 2: SYBR Primer sequence for qPCR

	Forward	Reverse
**Genomic**		
**TSPO**	AGACACCCCGTTTCGTAGTG	GGCCTCTAACCGGCTTTGG
**MYOD1**	GGGGGCGCTATTAGAACAC	CAAACCAAGGGCGGACAG
**GATA2**	CCTGTAATTAACCGCCAGCTC	GGCAGAACCGACCACTCATC
**BCL2**	AGATAGAGTTTTGTCATGTTGTGC	GGTGGGTGTATTGCTTGAGG
**mRNA**		
**HDAC1**	AATTCTTGCGCTCCATCCGT	GTTTCACAGCACTTGCCACA
**HDAC2**	ACTACGACGGTGATATTGGAA	CTTCGGCAGTGGCTTTATGG
**DNMT1**	GCCAAACGGAAACCTCAGGA	AGCGGTCTAGCAACTCGTTC
**DNMT3a**	ACGACCAGGAATTTGACCCT	TCCACCTGAATGCCCAAGTC
**DNMT3b**	GGTTTGGCGATGGCAAGTTC	TCGCACCCTAGCTTTCTCCA
**FKBP5**	ATGCAGCGGGAAGAACAATG	TCCCAGGATTCTTTGGCCTTT
**FKBP4**	TGGACATCAGCCCCAAACAG	GTCCCCAATCATGGGCATCT
**DNAJB11**	TGCTTATGAGGTTCTGTCAGATAGT	CGAGGGGTTCCTCCAAACAT
**STK4**	AGACGGTACAGCTGAGGAAC	TACGCTGCCATAGGACCCTT
**CXCL8**	CACCGGAAGGAACCATCTCA	GGCAAAACTGCACCTTCACAC
**TSPO**	CTACTCAGCCATGGGGTACG	GTGAAGCCTCCCAGCTCTTT
**DNM1**	GGAGCTAATCAGCACCGTTAGA	AGCATGACCTGCTCCTTAGT
**YWHAZ**	ACTTGACATTGTGGACATCGGA	GTGGGACAGCATGGATGACA
**GSK3B**	GACTAAGGATTCGTCAGGAACA	TACTCCAGCAGACGGCTACA
**CBL**	CGGAAGCACGTTCAGTCTGG	TTTTGGCACAGGAAGAGGTCTGG
**FYN**	AGCAGGATGCTGATCTAAACG	TCTAAATTCCAAAATGTCCGCCAAC
**CSF2RB**	CCACGGCCAATACATCGTCT	GGGCCATCTGGATGTTCACT
**INPP5D**	ACACCAAGCAGAAAGCGACA	CGCTGGTACTGCCATAAGACT
**CD86**	AGCCAAAATGGATCCCCAGTG	ATCTTCAGAGGAGCAGCACCAGA
**HSPA5**	GCTCAACATGGATCTGTTCCG	GAGTCGAGCCACCAACAAGA
**IFR8**	CAGCTCCTTCCAGACTGGTG	TCTGGGAGAATGCTGAATGGT
**APRT**	CTGGAGTACGGGAAGGCTGA	ACAGGCAGCGTTCATGGTT
**TCIRG2**	ATCACGGACTGCTTCCACTG	CTCAGGAACCGCTCTGTCTC
**RPL10**	TTCGGTGTGCCACTGAAGAT	TTCCGCCCCAGGTCAAAAAT
**FCER1G**	AGCAGTGGTCTTGCTCTTACT	TTTCGCACTTGGATCTTCAGT
**CD74**	CTGGAAGGTCTTTGAGAGCTG	TCTTCCTGGCACTTGGTCAG
**PSMB8**	CTCCTGGCTGACTTCTAGTCT	CTGAACGTTCCTTTCTCCGT
**MCM3**	AATGCCTCAAGTACACACTCCA	GATTGACTGCGCATGAGCTT
**ACLY**	GGATTTTGCGGGGTTCGTC	GACATGGCTGCAGAGAGACC
**CYP1A1**	GAACCTTCCCTGATCCTTGTGA	GTGTAGGGATCTTGGAGGTG
**MPO**	CCTGGTTAGCAGAGCTGGAC	GGGCCCATAAGTCAACCACA
**SPARC**	TGGCAGAGGTGACTGAGGTAT	CGTGTTTGCAGTGGTGGTTC
**RASSF5**	GCAGGATCCCAGAGT	GGTGAATTTACAGTTAGTGC
**ELMO1**	GCCGACCCAGAGTGTATAG	TATGGCCACCTTGACGATGT
**GNB2L1**	CTTCTGGAGGCAAGGATGGC	GATGTCCCCACCATCTAGCG
**TYK2**	GAAAGCCCTCAAGGCAGACT	GACTTCTCGCCTTGGTCCTC
**CD59**	ACCAGTTGGTGTAGGAGTTGA	TGCAGGCTATGACCTGAATGG
**NCAM1**	GGAGACCCCATTCCCTCCAT	CATGGTGCATGTACCTCTTGC
**TNFSF13B**	CACCGCGGGACTGAAAATCT	CTGCAATCAGTTGCAAGCAGT
