# Supplementary material for: The histone deacetylase inhibitor Romidepsin induces as a cascade of differential gene expression and altered histone H3K9 marks in myeloid leukaemia cells
Source: Oncotarget. 2019 May 28;10(37):3462–71. doi: 10.18632/oncotarget.26877 (PMC6544403; doi:10.18632/oncotarget.26877)
Supplement: Supplementary file 2 [file oncotarget-10-3462-s002.docx]

**Supplementary Table 1: 130 genes showed an overlap between microarray data and increased acetylation**

| Gene Name | Probe Set ID | Gene Title | Chromosomal Location |
| --- | --- | --- | --- |
| ABHD14B | 224821_at | abhydrolase domain containing 14B | chr3p21.2 |
| ACLY | 210337_s_at | ATP citrate lyase | chr17q21.2 |
| ADAMTSL4 | 226071_at | ADAMTS-like 4 | chr1q21.3 |
| AGPAT1 | 32836_at | 1-acylglycerol-3-phosphate O-acyltransferase 1 | chr6p21.3 |
| AKR7A2 | 214259_s_at | aldo-keto reductase family 7, member A2 (aflatoxin aldehyde reductase) | chr1p36.13 |
| ANKRD35 | 231118_at | ankyrin repeat domain 35 /// ankyrin repeat domain-containing protein 35-like | chr1q21.1 |
| ANXA6 | 244250_at | annexin A6 | chr5q33.1 |
| AP2M1 | 200613_at | adaptor-related protein complex 2, mu 1 subunit | chr3q28 |
| ARAP3 | 235806_at | ArfGAP with RhoGAP domain, ankyrin repeat and PH domain 3 | chr5q31.3 |
| ARHGAP18 | 225173_at | Rho GTPase activating protein 18 | chr6q22.33 |
| ARHGAP30 | 243433_at | Rho GTPase activating protein 30 | chr1q23.3 |
| ATP8B2 | 226771_at | ATPase, aminophospholipid transporter, class I, type 8B, member 2 | chr1q21.3 |
| ATP8B4 | 220416_at | ATPase, class I, type 8B, member 4 | chr15q21.2 |
| C19orf54 | 222052_at | chromosome 19 open reading frame 54 | chr19q13.2 |
| C1orf106 | 219010_at | chromosome 1 open reading frame 106 | chr1q32.1 |
| CASP1 | 211368_s_at | caspase 1, apoptosis-related cysteine peptidase | chr11q23 |
| CBR4 | 244052_at | carbonyl reductase 4 | chr4q32.3 |
| CCNB1IP1 | 217988_at | cyclin B1 interacting protein 1, E3 ubiquitin protein ligase | chr14q11.2 |
| CD37 | 238321_at | CD37 molecule | chr19q13.3 |
| CD59 | 228748_at | CD59 molecule, complement regulatory protein | chr11p13 |
| CD74 | 209619_at | CD74 molecule, major histocompatibility complex, class II invariant chain | chr5q32 |
| CDK5RAP3 | 218740_s_at | CDK5 regulatory subunit associated protein 3 | chr17q21.32 |
| CNPY2 | 209797_at | canopy 2 homolog (zebrafish) | chr12q15 |
| COX16 | 223191_at | COX16 cytochrome c oxidase assembly homolog (S. cerevisiae) | chr14q24.2 |
| CPVL | 208146_s_at | carboxypeptidase, vitellogenic-like | chr7p15.1 |
| CSF2RB | 205159_at | colony stimulating factor 2 receptor, beta, low-affinity (granulocyte-macrophage) | chr22q13.1 |
| CUEDC2 | 218097_s_at | CUE domain containing 2 | chr10q24.32 |
| CX3CR1 | 205898_at | chemokine (C-X3-C motif) receptor 1 | chr3p21.3 |
| CXorf57 | 219355_at | chromosome X open reading frame 57 | chrXq22.3 |
| CYP1A1 | 205749_at | cytochrome P450, family 1, subfamily A, polypeptide 1 | chr15q24.1 |
| CYP4F3 | 206515_at | cytochrome P450, family 4, subfamily F, polypeptide 3 | chr19p13.2 |
| DDIT4 | 202887_s_at | DNA-damage-inducible transcript 4 | chr10q22.1 |
| DENND3 | 212975_at | DENN/MADD domain containing 3 | chr8q24.3 |
| DGAT2 | 226064_s_at | diacylglycerol O-acyltransferase 2 | chr11q13.5 |
| EBP | 231320_at | emopamil binding protein (sterol isomerase) | chrXp11.23-p11.22 |
| ERP44 | 208959_s_at | endoplasmic reticulum protein 44 | chr9q31.1 |
| ESX1 | 1552445_a_at | ESX homeobox 1 | chrXq22.1 |
| F11R | 224097_s_at | F11 receptor | chr1q21.2-q21.3 |
| FAM53C | 218023_s_at | family with sequence similarity 53, member C | chr5q31 |
| FBXW11 | 209456_s_at | F-box and WD repeat domain containing 11 | chr5q35.1 |
| FCER1G | 204232_at | Fc fragment of IgE, high affinity I, receptor for; gamma polypeptide | chr1q23 |
| FKBP5 | 224856_at | FK506 binding protein 5 | chr6p21.31 |
| FTL | 213187_x_at | ferritin, light polypeptide | chr19q13.33 |
| FTO | 209702_at | fat mass and obesity associated | chr16q12.2 |
| FYB | 227266_s_at | FYN binding protein | chr5p13.1 |
| GM2A | 35820_at | GM2 ganglioside activator | chr5q33.1 |
| GPX1 | 200736_s_at | glutathione peroxidase 1 | chr3p21.3 |
| GRN | 216041_x_at | granulin | chr17q21.32 |
| HCP5 | 206082_at | HLA complex P5 (non-protein coding) | chr6p21.3 |
| HNMT | 228772_at | histamine N-methyltransferase | chr2q22.1 |
| IDS | 236823_at | iduronate 2-sulfatase | chrXq28 |
| IL4R | 203233_at | interleukin 4 receptor | chr16p12.1-p11.2 |
| INPP5D | 203332_s_at | inositol polyphosphate-5-phosphatase, 145kDa | chr2q37.1 |
| IP6K2 | 223165_s_at | inositol hexakisphosphate kinase 2 | chr3p21.31 |
| IRF8 | 204057_at | interferon regulatory factor 8 | chr16q24.1 |
| KIF21B | 204411_at | kinesin family member 21B | chr1q32.1 |
| LIN28B | 229349_at | lin-28 homolog B (C. elegans) | chr6q21 |
| LTA4H | 208771_s_at | leukotriene A4 hydrolase | chr12q22 |
| LY86 | 205859_at | lymphocyte antigen 86 | chr6p25.1 |
| LY96 | 206584_at | lymphocyte antigen 96 | chr8q21.11 |
| MAP3K13 | 233508_at | mitogen-activated protein kinase kinase kinase 13 | chr3q27 |
| MAP7D1 | 217943_s_at | MAP7 domain containing 1 | chr1p34.3 |
| MEST | 202016_at | mesoderm specific transcript | chr7q32 |
| MORF4L2 | 243857_at | mortality factor 4 like 2 | chrXq22 |
| MPO | 203949_at | myeloperoxidase | chr17q23.1 |
| MRAS | 225185_at | muscle RAS oncogene homolog | chr3q22.3 |
| MT1X | 208581_x_at | metallothionein 1X | chr16q13 |
| MT2A | 212185_x_at | metallothionein 2A | chr16q13 |
| N4BP2L2 | 235547_at | NEDD4 binding protein 2-like 2 | chr13q13.1 |
| NAV1 | 242986_at | neuron navigator 1 | chr1q32.3 |
| NCF4 | 207677_s_at | neutrophil cytosolic factor 4, 40kDa | chr22q13.1 |
| NLRC5 | 226474_at | NLR family, CARD domain containing 5 | chr16q13 |
| NOLC1 | 211951_at | nucleolar and coiled-body phosphoprotein 1 | chr10q24.32 |
| OGT | 229787_s_at | O-linked N-acetylglucosamine (GlcNAc) transferase | chrXq13 |
| PAG1 | 227354_at | phosphoprotein associated with glycosphingolipid microdomains 1 | chr8q21.13 |
| PAPSS1 | 209043_at | 3'-phosphoadenosine 5'-phosphosulfate synthase 1 | chr4q24 |
| PARVG | 244229_at | parvin, gamma | chr22q13.31 |
| PCCB | 212694_s_at | propionyl CoA carboxylase, beta polypeptide | chr3q21-q22 |
| PCYOX1L | 218953_s_at | prenylcysteine oxidase 1 like | chr5q32 |
| PEA15 | 200788_s_at | phosphoprotein enriched in astrocytes 15 | chr1q21.1 |
| PECAM1 | 208983_s_at | platelet/endothelial cell adhesion molecule 1 | chr17q23.3 |
| PIH1D1 | 217872_at | PIH1 domain containing 1 | chr19q13.33 |
| PNPO | 222653_at | pyridoxamine 5'-phosphate oxidase | chr17q21.32 |
| POC1A | 234749_s_at | POC1 centriolar protein homolog A (Chlamydomonas) | chr3p21.2 |
| PRAME | 204086_at | preferentially expressed antigen in melanoma | chr22q11.22 |
| PRIM1 | 205053_at | primase, DNA, polypeptide 1 (49kDa) | chr12q13 |
| PRPS1 | 209440_at | phosphoribosyl pyrophosphate synthetase 1 | chrXq22.3 |
| PRRC2A | 214201_x_at | proline-rich coiled-coil 2A | chr6p21.3 |
| PRRC2B | 223793_at | proline-rich coiled-coil 2B | chr9q34.13 |
| PSAP | 200871_s_at | prosaposin | chr10q21-q22 |
| PSMD3 | 201388_at | proteasome (prosome, macropain) 26S subunit, non-ATPase, 3 | chr17q21.1 |
| QPRT | 242414_at | quinolinate phosphoribosyltransferase | chr16p11.2 |
| RAB8B | 226633_at | RAB8B, member RAS oncogene family | chr15q22.2 |
| RAD51 | 205024_s_at | RAD51 homolog (S. cerevisiae) | chr15q15.1 |
| RASSF4 | 49306_at | Ras association (RalGDS/AF-6) domain family member 4 | chr10q11.21 |
| RASSF5 | 223322_at | Ras association (RalGDS/AF-6) domain family member 5 | chr1q32.1 |
| RDH11 | 232359_at | retinol dehydrogenase 11 (all-trans/9-cis/11-cis) | chr14q24.1 |
| RGS16 | 242212_at | regulator of G-protein signaling 16 | chr1q25-q31 |
| RNF114 | 211678_s_at | ring finger protein 114 | chr20q13.13 |
| RNF135 | 223592_s_at | ring finger protein 135 | chr17q11.2 |
| RNFT2 | 221909_at | ring finger protein, transmembrane 2 | chr12q24.22 |
| SASH1 | 41644_at | SAM and SH3 domain containing 1 | chr6q24.3 |
| SAT1 | 213988_s_at | spermidine/spermine N1-acetyltransferase 1 | chrXp22.1 |
| SFXN3 | 220974_x_at | sideroflexin 3 | chr10q24.31 |
| SLPI | 203021_at | secretory leukocyte peptidase inhibitor | chr20q12 |
| SNRPA | 201770_at | small nuclear ribonucleoprotein polypeptide A | chr19q13.1 |
| SP110 | 223980_s_at | SP110 nuclear body protein | chr2q37.1 |
| SPATA20 | 218164_at | spermatogenesis associated 20 | chr17q21.33 |
| SQRDL | 217995_at | sulfide quinone reductase-like (yeast) | chr15q15 |
| STK4 | 243981_at | serine/threonine kinase 4 | chr20q11.2-q13.2 |
| SYTL1 | 227134_at | synaptotagmin-like 1 | chr1p36.11 |
| TBC1D7 | 223461_at | TBC1 domain family, member 7 | chr6p24.1 |
| TCEAL3 | 227279_at | transcription elongation factor A (SII)-like 3 | chrXq22.2 |
| TIFA | 238858_at | TRAF-interacting protein with forkhead-associated domain | chr4q25 |
| TMEM161A | 43977_at | transmembrane protein 161A | chr19p13.11 |
| TNFSF13B | 223502_s_at | tumor necrosis factor (ligand) superfamily, member 13b | chr13q32-q34 |
| TTC7B | 226152_at | tetratricopeptide repeat domain 7B | chr14q32.11 |
| TUBA4A | 212242_at | tubulin, alpha 4a | chr2q35 |
| TUBG1 | 201714_at | tubulin, gamma 1 | chr17q21 |
| TYK2 | 205546_s_at | tyrosine kinase 2 | chr19p13.2 |
| UCP2 | 208998_at | uncoupling protein 2 (mitochondrial, proton carrier) | chr11q13 |
| UGGT1 | 244814_at | UDP-glucose glycoprotein glucosyltransferase 1 | chr2q14.3 |
| UROS | 232560_at | uroporphyrinogen III synthase | chr10q25.2-q26.3 |
| VARS2 | 226200_at | valyl-tRNA synthetase 2, mitochondrial | chr6p21.33 |
| VIPR1 | 205019_s_at | vasoactive intestinal peptide receptor 1 | chr3p22 |
| WDFY4 | 229597_s_at | WDFY family member 4 | chr10q11.23 |
| ZMYND8 | 230533_at | zinc finger, MYND-type containing 8 | chr20q13.12 |
| ZNF217 | 203739_at | zinc finger protein 217 | chr20q13.2 |
